# Geometric and Dosimetric Changes in Tumor and Lung Tissue During Radiotherapy for Lung Cancer With Atelectasis

**DOI:** 10.3389/fonc.2021.690278

**Published:** 2021-07-22

**Authors:** Hua Chen, Yan Shao, Xiaohua Gu, Zhijie Zheng, Hao Wang, Hengle Gu, Yanhua Duan, Aihui Feng, Ying Huang, Wutian Gan, Chongyang Chen, Zhiyong Xu

**Affiliations:** ^1^ Institute of Modern Physics, Fudan Univerisity, Shanghai, China; ^2^ Shanghai Chest Hospital, Shanghai Jiaotong University, Shanghai, China; ^3^ School of Physical Science and Technology, Wuhan University, Wuhan, China

**Keywords:** atelectasis, lung cancer, cone beam computed tomography (CBCT), center of mass, radiotherapy, regression, dosimetry comparison

## Abstract

**Background and Purpose:**

This article retrospectively characterized the geometric and dosimetric changes in target and normal tissues during radiotherapy for lung cancer patients with atelectasis.

**Materials and Methods:**

A total of 270 cone beam computed tomography (CBCT) scans of 18 lung patients with atelectasis were collected. The degree and time of resolution or expansion of the atelectasis were recorded. The geometric, dosimetric, and biological changes in the target and lung tissue were also quantified.

**Results:**

There were two patients with expansion, four patients with complete regression, six patients with partial regression, and six patients with no change. The time of resolution or expansion varied. The tumor volume increased by 3.8% in the first seven fractions, then decreased from the 9th fraction, and by 33.4% at the last CBCT. In the LR direction, the average center of mass (COM), boundaries of the tumors gradually shifted mediastinally. In the AP direction, the COM of the tumors was shifted slightly in the posterior direction and then gradually shifted to the anterior direction; the boundaries of the tumors all moved mediastinally. In the SI direction, the COM of the tumors on the right side of the body was substantially shifted toward the head direction. The boundaries of the tumors varied greatly. D_2_, D_98_, D_mean_, V_95_, V_107_, and TCP of the PTV were reduced during radiotherapy and were reduced to their lowest values during the last two fractions. The volume of the ipsilateral lung tended to increase gradually. The V_5_, V_10_, V_20_, V_30_, V_40_, and NTCP of the total lung gradually increased with the fraction.

**Conclusions:**

For most patients, regression of the atelectasis occurred, and the volume of the ipsilateral lung tended to increase while the tumor volume decreased, and the COM and boundary of the tumors shifted toward mediastinum, which caused an insufficient dose to the target and an overdose to the lungs. Regression or expansion may occur for any fraction, and it is therefore recommended that CBCT be performed at least every other day.

## Introduction

Lung cancer is one of the most common malignant cancers, with high rates of morbidity and mortality around the world, and non-small cell lung cancer makes up the majority of lung cancer cases (1, 2). Some studies have indicated a 10–40% incidence of atelectasis being present at the beginning of lung cancer radiotherapy treatment (3–7). Atelectasis is a primary malignant change of the bronchial mucosa epithelium, which forms a polypoid mass in the lumen, blocking the lumen directly or indirectly by compression of external lesions, resulting in a reduction in lung capacity, thus inducing atelectasis. Atelectasis often occurs in central lung cancer (8–10).

The regression or progression of lung tumors during radiotherapy may lead to the regression or expansion of atelectasis, and this anatomical change will cause deviations in the tumor location and dosimetric changes not reflected in the planning CT (3–6, 11–13), which cannot be solved by increasing the safety margin (14, 15) so a more individualized adaptive strategy is needed. Additionally, the dose to the target volume and the normal tissue are also altered, so the introduction of image-guided radiotherapy technology is very important for lung cancer patients with atelectasis (16, 17).

At present, there are few reports on atelectasis during radiotherapy course. Nathan Tennyson et al. studied the variation in atelectasis volumes and the effect of atelectasis volume changes on the primary tumor position during radiation therapy (12), but the dosimetric effects on the target volume or surrounding organs were not considered. Some data showed that the changes in the mass and density of atelectasis during radiotherapy could also cause dosimetric effects on the normal tissue structure (11). Moller et al. (5), based on weekly cone beam computed tomography (CBCT), found that 70% of 24 patients with atelectasis need adaptive radiotherapy due to geometric shifts and/or dosimetric changes of the tumor caused by atelectasis and that atelectasis appeared/disappeared in 22% of the patients at the first treatment. The above studies provide important data about the changes of atelectasis volumes and the effect on the geometric and dosimetric changes to the tumor and surrounding normal tissues during radiotherapy. However, the geometric (volume and location) changes in the tumor and lung tissue caused by the regression or expansion of atelectasis during radiotherapy have not been well defined, and the dosimetric effect of the target and lung has not been clarified.

In this paper, we studied lung cancer patients with atelectasis who underwent CBCT before radiotherapy to evaluate the regularity of geometric and dosimetric changes in the target volume and lung tissue during radiotherapy and to perform preliminary clinical data analysis for a study of adaptive radiotherapy in patients with atelectasis.

## Methods and Materials

### Patient Information

Twenty-five patients with lung cancer stages IIA to IIIB who were treated with thoracic radiotherapy between January 2019 and December 2019 at our center were included in this study. All patients were found to have atelectasis before radiotherapy. Among them, five patients had to be excluded due to early termination of radiotherapy, and two patients were excluded because of incomplete data. A total of 18 patients were included in this study. The patient characteristics are shown in **Table 1**.

**Table 1:** Patient characteristics.

Patients Group	n = 18
**Gender**	
Male	17
Female	1
**Median Age**	65(range52–77)
**Tumor Type**	
Central	15
Peripheral	3
**Tumor Stage**	
T1–2	3
T3–4	15
**Nodal Stage**	
N0–1	2
N2–3	16
**Atelectasis Site**	
RUL	7
RDL	4
LDL	1
LUL	6
Whole left lung	2
**GTV Volume (cm^3^)**	
Mean	197.21
Range	27.93–618

### Contouring and Treatment Planning

Patient simulation occurred after the patient was immobilized with a thermoplastic mask or vacuum cushion in the supine position. CT scans were performed using a Siemens Somatom Definition AS CT Scanner System (Siemens Healthcare, Erlangen, Germany) under free breathing conditions. The patient was scanned from cervical vertebrae C3 to the lower edge of the liver, including the entire lung, with a slice thickness of 3 mm. The CT data of each patient were transferred to the Pinnacle^3^ treatment planning system v9.10 (Philips Healthy, Fitchburg, WI).

The target volumes and organs at risk (OARs) of each patient were delineated on the Pinnacle^3^ treatment planning system. The gross tumor volume (GTV) included both the primary tumor and pathologically proven lymph nodes. When available, contrast-enhanced CT scans and 18-FDG positron emission tomography (PET)-CT scans were used to distinguish GTV from atelectasis during contouring. The planning target volume (PTV) expanded an isotropic 5 mm margin on GTV to account for set-up uncertainties and respiratory motion. All contours for the tumors were peer reviewed to ensure accuracy and reproducibility. OARs included the total lung, bronchi, spinal cord, and heart. Total lung was defined as the lung volume minus the GTV.

Treatment plans were performed on the Pinnacle treatment planning system with four to eight 6 MV photon beams, and the dose was delivered using a Synergy linear accelerator (Elekta, Crawley, UK), which had an MLCi2 with 80 leaves. A total of 60 Gy in 30 fractions was prescribed for PTV. The optimization goals were to deliver the prescription dose to at least 95% of the PTV.

### CBCT Scans and CT Scans

CBCT scans were performed for every two fractions for each patient, and a total of 15 CBCT scans were obtained at the end of radiotherapy. A rigid registration was performed online based on the bony anatomy of the spine visible on the initial planning CT scan to correct for daily setup errors at each treatment fraction. If obvious visible regression or expansion of the atelectasis was found, a 3D-CT scan and a contrast-enhanced CT scan were performed, and then dosimetric evaluation was performed to create adaptive treatment plans if needed. The patients underwent a median of three CT scans (including conventional CT scans and contrast-enhanced CT scans) ranging from two to four during the radiation therapy.

The geometric and dosimetric changes of each CBCT image relative to the planning CT were assessed offline. All CBCT and CT scans were transferred to commercial radiation oncology software MIM (MIM Maestro v6.6.4, Cleveland, OH, USA), and rigid registration was performed with the planning CT based on the bony anatomy of the spine. The tumor, atelectasis, and ipsilateral lung were contoured with lung and soft tissue windows in each CBCT image. All contours were drawn by an experienced radiation oncologist and reviewed by another radiation oncologist for accuracy and consistency.

To evaluate the geometric change of atelectasis (regression or expansion) and its effect on the tumor and volume, center of mass (COM) and boundary shift changes of the tumor were recorded, and the volume changes of the ipsilateral lung were also noted. The fraction number of atelectasis regressions or progression was also recorded. The boundary change is defined as the six border position changes compared with the initial planning CT scan and calculated in the left–right (LR), anterior–posterior (AP), and superior–inferior (SI) directions.

As the resolution of CBCT images is limited, only a change in volume of more than 10% is considered significant. Compared to the planning CT, if the atelectasis volume reduction on CBCT was less than 10%, it was labeled as no regression; if a decrease in volume was between 10 and 90%, it was labeled as partial regression; if the reduction was more than 90%, it was labeled as complete regression; if the increased atelectasis volume was more than 10%, it was labeled as expansion.

### Dosimetric Evaluation

The regression or expansion of atelectasis may change the density, size, and anatomical position of the lung tissue and the anatomical position of the tumor during the treatment course, which will lead to insufficient target coverage or an overdose to the lung tissue. The dosimetric change was evaluated between the CBCT/CT scan and the planning CT scan.

The contours of the tumor and ipsilateral lung were drawn on the CBCT scans, and then the structures were cropped to the initial planning CT scan. The Hounsfield unit (HU) of the lung with density changes was set to lung (HU = −738 corresponding to 0.26 g/cm^3^) or water (HU = 0) on the planning CT scan according to whether the density change of the lung structure disappeared or reappeared on the CBCT image (5). We assumed that the contralateral lung volume remained constant during treatment; therefore, the total lung volume was defined as the new ipsilateral lung volume plus the contralateral lung volume, which was drawn on the initial planning CT scan. The dose was recalculated at the planning CT with alteration of the HU.

For the CT scans, the contours of the tumor and ipsilateral and contralateral lungs were drawn. The dose was recalculated based on the CT scan instead of the planning CT scan.

The dose distribution obtained from the altered CT scan was compared with the planned dose distribution of the planning CT scan. The dosimetric evaluation parameters included D_2_, D_98_, D_mean_, V_95_, and V_107_ of the PTV and V_5_, V_10_, V_20_, V_30_, and V_40_ of the total lung.

### Biological Evaluation

The tumor control probability (TCP) of the PTV and normal tissue complication probability (NTCP) of total lung were calculated. Both the TCP and NTCP calculations were performed on MATLAB R2019a (The MathWorks Inc., MA, USA). Based on the following equations (18), the TCP were calculated as follows:

$$TCP=\frac{1}{1+{\left(\frac{TCD_{50}}{EUD}\right)}^{4\gamma_{50}}}$$

$$EUD={\left[\sum_{i=1}\left(v_{i}*D_{i}^{a}\right)\right]}^{\frac{1}{\alpha }}$$

where TCD_50_ was the tumor dose required to produce a 50% TCP, *γ*_50_ was the change in TCP expected because of a 1% change in TCD_50_. D*_i_* was a uniform dose of partial volume V_i_ (19). The values of TCD_50_, *γ*_50_ and *α* were 51.24 Gy, 0.83 and 0.30, respectively.

The NTCP was calculated basing on the Lyman–Kutcher–Burman model (20). The equations were as follows:

$$\text{NTCP}=\frac{1}{\sqrt{2\pi }}\int_{-\infty }^{t}{\text{e}}^{-\frac{x^{2}}{2}}\text{d}x$$

$$t=\frac{{\text{D}}_{\text{eff}}-T{\text{D}}_{50}}{m*TD_{50}}$$

$${\text{D}}_{\text{eff}}={\left(\sum_{i}v_{i}*{\text{D}}_{i}^{1/n}\right)}^{n}$$

For pneumonia, the TD_50_, n and m published by Semenko (21) were 29.9 Gy, 1 and 0.41, respectively.

### Statistical Analysis

All of the parameters are reported as the mean ± standard deviation. The statistical analysis was performed using SPSS Statistics v22.0 software (IBM Corp., Armonk, NY, USA). A paired, two-sided Wilcoxon signed-rank test was used to evaluate the differences in volume, COM, boundary shifts of the tumor, volume of the ipsilateral lung, dosimetric parameters of the PTV, and total lung between the CBCT/CT scans and the planning CT scan. Statistical results were considered statistically significant at p <0.05.

## Results

**Figure 1:**
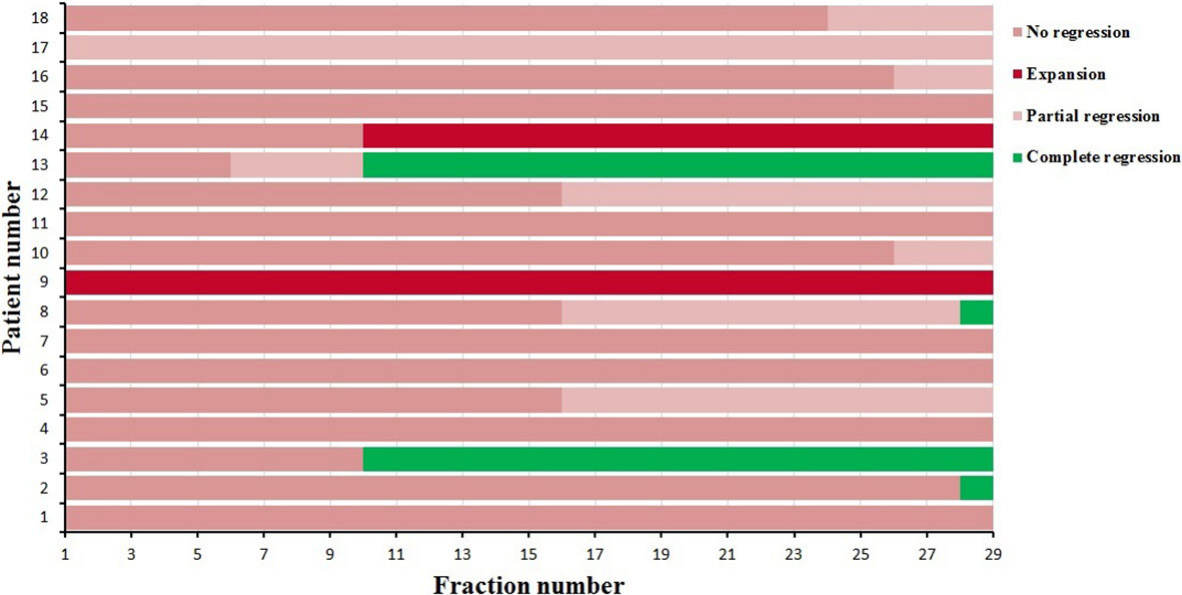
The degree and time atelectasis regression or expansion for 18 patients during radiotherapy.

The degree and fraction number of atelectasis regressions or expansions that occurred during radiotherapy were recorded. Four of the 18 patients had complete regression during radiotherapy, two patients had progression, six patients had partial regression and were not fully regressed at the end of radiotherapy, and the remaining six had almost no change. As shown in **Figure 1**, the time of regression or expansion varied for each patient. Thus, the results of the analysis of the 270 CBCT images acquired in this study showed that in addition to two patients with atelectasis progression and four patients with no change, the remaining twelve patients with atelectasis underwent regression.

### The Volume Changes of the GTV

The initial mean volume of the GTV was 197.29 cm^3^ (27.93–618 cm^3^). During the treatment course, the volume of the GTV increased slowly in the first seven fractions and gradually decreased from the ninth fraction (see **Table 2**).

**Table 2:** The volume changes of GTV.

		GTV Volume	GTV (≤150 cm^3^)		GTV (>150 cm^3^)	
			Volume (cm^3^)	Change (%)	Volume (cm^3^)	Change (%)
Plan		197.29 ± 153.8	103.58 ± 38.07	–	344.55 ± 152.32	–
f1		200.86 ± 157.22	102.77 ± 37.01	−0.72 ± 4.87	355.01 ± 149.62	3.77 ± 5.33
f3		203.29 ± 161.39	102.15 ± 36.32	−0.92 ± 6.6	362.21 ± 152.93	6.21 ± 10.14
f5		206.99 ± 168.12	101.34 ± 36	−1.46 ± 8.61	373.01 ± 159.08	9.68 ± 16.62
f7		200.18 ± 158.47	99.45 ± 35.74	−3.04 ± 9.5	358.47 ± 145.56	5.81 ± 12.63
f9		191.74 ± 151.83	98.92 ± 34.46	−5.22 ± 11.09	337.59 ± 150.74	−1.8 ± 16.9
f11		172.69 ± 136.34	93.49 ± 31.08	−6.48 ± 14.97	297.14 ± 146.6	−14.27 ± 26.44
f13		168.09 ± 133.95	90.95 ± 29.98	−8.53 ± 16.54	289.31 ± 145.9	−16.88 ± 27.1
f15		158.45 ± 125.33	88.48 ± 30.95	−11.47 ± 16.41	268.4 ± 140.82	−22.81 ± 29.94
f17		148.57 ± 118.93	84.43 ± 30.27	−15.29 ± 17.1	249.36 ± 138.4	−28.77 ± 29.53
f19		146.95 ± 116.64	85.48 ± 31.98	−14.47 ± 18.16	243.55 ± 137.94	−31.03 ± 28.64
f21		146.65 ± 111.98	88.07 ± 33.69	−12.68 ± 16.37	238.71 ± 132.12	−31.34 ± 29.48
f23		141.44 ± 110.42	84.23 ± 31.95	−15.94 ± 17.07	231.34 ± 131.98	−33.43 ± 29.47
f25		138.75 ± 108.28	83.14 ± 31.43	−16.85 ± 17.38	226.15 ± 130.35	−35.29 ± 28.67
f27		133.6 ± 102.52	82.28 ± 35.53	−21.35 ± 18.09	214.25 ± 123.54	−38.84 ± 26.22
f29		131.25 ± 97.77	81.75 ± 34.72	−21.75 ± 17.09	209.04 ± 116.28	−39.68 ± 26.23

**Figure 2:**
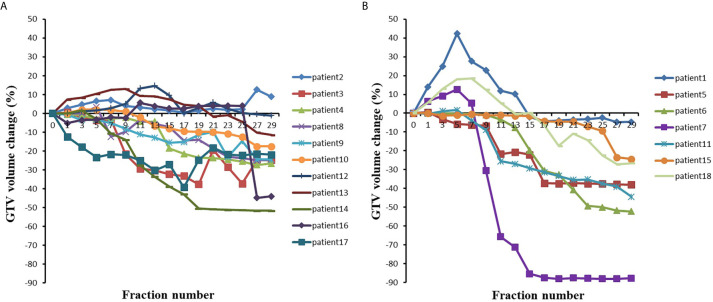
The GTV volumes changes with the fraction for **(A)** 11 patients with initial GTV volume less than 150 cm^3^ and **(B)** 7 patients with initial GTV volume greater than 150 cm^3^.

For the eleven patients with an initial GTV volume less than 150 cm^3^, the mean GTV volume tended to gradually decrease during the course of radiotherapy, reaching a 5.22% reduction in GTV volume at the 7th fraction and a 21.75% reduction in GTV volume at the end of treatment. As shown in **Figure 2A**, the GTV volume continued to decrease in five patients and became larger in one patient, but in five patients, the GTV volume first gradually increased and then slowly decreased.

For the seven patients with an initial GTV volume greater than 150 cm^3^, the mean GTV volume gradually increased to 5.81% during the first seven fractions and began to decrease by the 9th fraction, and the mean GTV volume had decreased by 39.68% at the end of radiotherapy. In three of these seven patients, the GTV volume gradually increased during the first seven fractions (see **Figure 2**) and essentially decreased from the ninth fraction. The remaining four patients showed a gradual decrease in the GTV.

As shown in **Figure 2** and **Table 2**, the GTV volume changed greatly during radiotherapy, regardless of whether the volume decreased or increased. Most of these changes were caused by atelectasis regression or expansion. If change of GTV volume was large, the collected CBCT image and the original CT may not be registered, which indicated the necessity of adaptive radiotherapy.

### The COM Changes of the GTV

The COM of the GTV was located on the right side of the body in nine of the 18 patients, and the COM of the GTV was located on the left side of the body in nine of the patients.

**Figure 3:**
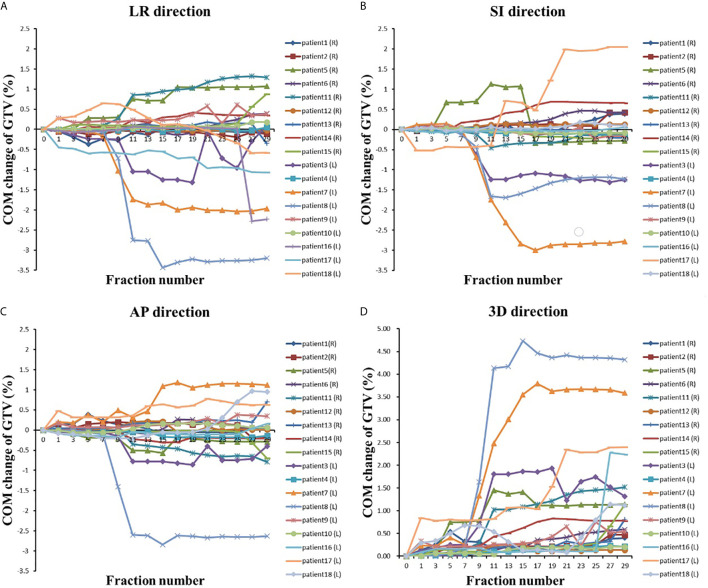
The COM changes with the fraction in **(A)** LR direction, **(B)** AP direction, **(C)** LR direction and **(D)** 3D direction.

In the LR direction, the mean COM of the GTV on the right side of the body was not shifted more than 0.5 cm during the treatment course (see **Table 3**). The COM was shifted slightly to the right during the first few treatment fractions and then gradually shifted to the left (*i.e.*, toward the mediastinal direction). The COM of the GTV on the left side of the body was gradually shifted to the right (mediastinal direction), and the shift exceeded 0.5 cm at the 11th treatment fraction and reached the maximum average shift (1 cm) at the 27th treatment fraction. In general, the COM of the GTVs gradually shifted toward the mediastinum, as shown in **Figure 3**, and the COM shifts of the GTV on the right side of the body were much smaller than those of the GTV on the left side of the body throughout the entire radiotherapy process.

**Table 3:** The COM shifts of GTV.

	COM shifts of GTV on the right side of body				COM shifts of GTV on the left side of body			
	X (cm)	Y (cm)	Z (cm)	3D (cm)	X (cm)	Y (cm)	Z (cm)	3D (cm)
f1	−0.02 ± 0.03	0.03 ± 0.04	−0.01 ± 0.02	0.05 ± 0.05	0.00 ± 0.21	0.07 ± 0.18	−0.04 ± 0.19	0.21 ± 0.26
f3	−0.03 ± 0.08	0.01 ± 0.08	−0.01 ± 0.03	0.09 ± 0.07	−0.02 ± 0.22	0.03 ± 0.14	−0.02 ± 0.20	0.22 ± 0.23
f5	−0.01 ± 0.18	0.03 ± 0.16	0.06 ± 0.23	0.21 ± 0.26	−0.03 ± 0.29	0.03 ± 0.19	−0.03 ± 0.16	0.28 ± 0.25
f7	0.01 ± 0.14	0.03 ± 0.13	0.10 ± 0.22	0.21 ± 0.22	−0.02 ± 0.33	0.0 0.17	−0.07 ± 0.15	0.30 ± 0.26
f9	0.04 ± 0.17	0.02 ± 0.12	0.11 ± 0.24	0.24 ± 0.22	−0.20 ± 0.51	−0.12 ± 0.53	−0.26 ± 0.28	0.62 ± 0.57
f11	0.22 ± 0.34	−0.06 ± 0.25	0.14 ± 0.42	0.43 ± 0.48	−0.59 ± 1.07	−0.32 ± 0.92	−0.57 ± 0.76	1.14 ± 1.41
f13	0.21 ± 0.34	−0.07 ± 0.26	0.18 ± 0.39	0.46 ± 0.44	−0.62 ± 1.07	−0.26 ± 0.97	−0.53 ± 0.98	1.22 ± 1.49
f15	0.22 ± 0.37	−0.09 ± 0.28	0.16 ± 0.40	0.46 ± 0.48	−0.74 ± 1.23	−0.21 ± 1.12	−0.57 ± 1.09	1.34 ± 1.72
f17	0.25 ± 0.46	−0.05 ± 0.24	0.00 ± 0.30	0.47 ± 0.42	−0.76 ± 1.22	−0.19 ± 1.06	−0.59 ± 1.09	1.34 ± 1.69
f19	0.27 ± 0.46	−0.06 ± 0.25	0.03 ± 0.32	0.48 ± 0.44	−0.73 ± 1.21	−0.20 ± 1.05	−0.49 ± 1.16	1.39 ± 1.63
f21	0.30 ± 0.48	−0.05 ± 0.27	0.07 ± 0.32	0.52 ± 0.46	−0.62 ± 1.26	−0.11 ± 1.07	−0.39 ± 1.31	1.43 ± 1.66
f23	0.32 ± 0.50	−0.10 ± 0.26	0.09 ± 0.32	0.54 ± 0.47	−0.76 ± 1.17	−0.13 ± 1.08	−0.37 ± 1.31	1.45 ± 1.65
f25	0.33 ± 0.51	−0.09 ± 0.26	0.10 ± 0.32	0.57 ± 0.46	−0.76 ± 1.22	−0.08 ± 1.12	−0.39 ± 1.30	1.56 ± 1.58
f27	0.40 ± 0.50	−0.15 ± 0.26	0.14 ± 0.34	0.65 ± 0.42	−1.00 ± 1.25	−0.04 ± 1.13	−0.39 ± 1.32	1.81 ± 1.49
f29	0.38 ± 0.58	−0.15 ± 0.44	0.14 ± 0.34	0.77 ± 0.43	−0.93 ± 1.28	0.011.11	−0.38 ± 1.31	1.77 ± 1.48

The COM shifts of the GTVs in the AP direction are shown in **Table 3**. The mean COM of the GTV on the right side of the body was first slightly shifted backward during radiotherapy and then gradually shifted forward, and the maximum shift was 0.15 cm by the last treatment fraction. As shown in **Figure 3**, the COM shift of the GTV on the right side of the body exceeded 0.5 cm in four patients. Among them, two patients also had a shift of more than 0.5 cm in the LR direction, and the other two patients had a shift of more than 0.5 cm in the COM only during the last treatment fraction. The mean COM of the GTV on the left side of the body also shifted slightly backward in the first seven treatment fractions, with the largest shift (0.32 cm) in the 11th radiotherapy session, after which the forward shift gradually decreased. As shown in **Figure 3**, similar to the COM shift in the LR direction, the COM shifts of GTV on the right side of body were smaller than that of the GTV on the left side of the body throughout the radiotherapy, and the COM shifts of the GTV on the left side of body exceeded 0.5 cm in five patients. All of them also had a shift of more than 0.5 cm in the LR direction at the same time.

The mean COM of the GTV on the right side of the body was basically shifted toward the head in the SI direction during the treatment course, and the shifts were small, no more than 0.2 cm. The mean COM position of the GTV on the left side of the body was shifted toward the foot, and the maximum shift was 0.59 cm. There were six patients with COM shifts exceeding 0.5 cm during radiotherapy, including four patients with GTV on the left side of the body and two patients with GTV on the right side of the body.

We also analyzed the mean COM shift of the GTV in the 3-D direction. As shown in **Table 3**, the COM shifts of all of the GTVs gradually increased during radiotherapy and almost reached a maximum by the last treatment fraction, which was 0.77 cm for the GTV on the right side and 1.81 cm for the GTV on the left side, 2.34 times more than that on the right side. The GTV with the COM shift was located on the left side of the body, and the shift was as high as 4.73 cm by the seventh treatment fraction. A total of 77.8% (14 patients) of the patients had a shift >0.5 cm, and 50% (nine patients) of the patients had a shift >1 cm.

### The Boundary Changes of the GTV

The changes in the GTV boundary position are shown in **Table 4**. In the LR direction, both the left and right boundaries of the GTV on the right side of the body were gradually shifted to the left, *i.e.*, toward the mediastinum, which was consistent with the direction of the GTV COM shift, and the average shift of the left boundary was larger than that of the right boundary, with a maximum of 0.86 cm. Similarly, the left and right boundaries of the GTV on the left side of the body were also shifted toward the mediastinal region, with a maximum shift of 1.29 cm.

**Table 4:** The boundary shifts of GTV on the left and right sides of body.

	Boundary shift of GTV on the right side of body (cm)						Boundary shift of GTV on the left side of body (cm)					
	Left boundary	Right boundary	Anterior boundary	Posterior boundary	Superior boundary	Inferior boundary	Left boundary	Right boundary	Anterior boundary	Posterior boundary	Superior boundary	Inferior boundary
f1	0.01±0.03	-0.01±0.03	0.00±0.00	0.18±0.41	0.00±0.00	0.00±0.00	0.07±0.14	0.06±0.00	0.13±0.40	0.17±0.54	-0.14±0.43	0.00±0.00
f3	-0.02±0.12	-0.01±0.03	0.00±0.00	0.13±0.46	0.00±0.00	-0.06±0.17	0.07±0.14	-0.03±0.44	0.13±0.40	0.10±0.34	-0.14±0.43	0.00±0.00
f5	-0.10±0.41	-0.01±0.06	-0.01±0.03	0.20±0.55	0.06±0.17	-0.06±0.17	0.07±0.14	0.07±0.67	0.04±0.42	0.12±0.45	-0.14±0.43	0.00±0.00
f7	-0.11±0.28	-0.08±0.52	-0.02±0.04	0.14±0.46	0.07±0.17	-0.06±0.17	0.10±0.2	0.07±0.65	0.01±0.44	0.07±0.44	-0.14±0.44	0.02±0.07
f9	-0.21±0.40	-0.08±0.13	-0.02±0.04	0.11±0.42	0.06±0.17	-0.06±0.17	0.14±0.41	-0.03±0.69	-0.21±0.92	-0.14±0.73	-0.33±0.61	0.18±0.53
f11	-0.34±0.56	-0.09±0.15	-0.13±0.19	-0.10±0.51	0.06±0.17	-0.06±0.17	-0.16±1.48	-0.56±1.45	-0.06±0.71	-0.36±1.25	-0.64±0.80	0.46±0.90
f13	-0.43±0.54	-0.07±0.14	-0.20±0.17	-0.10±0.51	0.06±0.17	-0.06±0.17	-0.36±1.75	-0.58±1.53	-0.11±0.64	-0.4±1.20	-0.61±1.28	0.52±0.98
f15	-0.49±0.58	-0.22±0.46	-0.26±0.38	-0.22±0.49	0.06±0.17	-0.11±0.22	-0.20±1.50	-1.11±1.72	-0.80±1.33	-0.23±1.85	-0.78±1.60	0.58±0.92
f17	-0.47±0.56	-0.23±0.46	-0.24±0.61	-0.22±0.6	-0.07±0.20	-0.12±0.24	-0.21±1.48	-1.17±1.85	-0.81±1.97	-1.00±1.57	-0.84±1.74	0.51±0.96
f19	-0.54±0.57	-0.2±0.48	-0.19±0.75	-0.27±0.49	-0.12±0.24	-0.18±0.27	-0.22±1.30	-0.86±1.96	-0.77±1.93	-1.13±1.51	-0.72±1.70	0.53±0.97
f21	-0.62±0.70	-0.21±0.5	-0.14±0.65	-0.40±0.75	-0.12±0.24	-0.18±0.27	-0.23±1.31	-1.07±1.78	-0.87±1.94	-0.94±1.43	-0.49±2.10	0.51±0.96
f23	-0.67±0.78	-0.13±0.39	-0.09±0.63	-0.41±0.76	-0.12±0.24	-0.12±0.24	-0.20±1.32	-1.18±1.72	-0.91±1.92	-1.06±1.60	-0.50±2.11	0.53±0.97
f25	-0.72±0.76	-0.11±0.4	-0.17±0.68	-0.42±0.75	-0.14±0.24	-0.18±0.27	-0.21±1.31	-1.29±1.69	-0.97±1.91	-1.08±1.57	-0.50±2.11	0.52±0.95
f27	-0.86±0.75	-0.29±0.65	-0.20±0.72	-0.51±0.68	-0.12±0.24	-0.19±0.28	-0.21±1.31	-1.18±1.78	-1.04±1.93	-1.03±1.46	-0.50±2.11	0.52±0.95
f29	-0.80±0.82	-0.11±0.38	-0.18±0.75	-0.53±0.67	-0.12±0.24	-0.18±0.27	-0.21±1.31	-1.09±1.81	-1.19±2.00	-0.98±1.38	-0.56±2.14	0.47±0.86

In the AP direction, the anterior boundary of the GTV on the right side of the body gradually shifted to the posterior; the posterior boundary had a tendency to shift to the anterior, and the shifts of both the anterior and posterior boundaries were not very large, with a maximum of 0.53 cm. The anterior boundary of the GTV on the left side of the body shifted to the anterior during the first seven fractions and gradually shifted to the posterior by the 9th fraction, while the posterior boundary shifted to the posterior during the first seven fractions and then gradually shifted to the anterior, and the shift of both boundaries was more than 1 cm by the end of radiotherapy. Therefore, the anterior and posterior boundaries of all of the GTVs had a tendency to shift toward the mediastinal region.

In the SI direction, the upper boundary of the GTV on the right side of the body was first shifted toward the head during the first 15 treatment fractions and then toward the foot. The lower boundary was gradually shifted toward the head throughout the treatment course. For the GTV on the right side of the body, both the upper and lower boundaries were shifted by no more than 0.2 cm. For the GTV on the left side of the body, both the anterior and posterior boundaries were gradually shifted toward the foot, and the shift of the upper boundary was slightly greater than that of the lower boundary.

### The Volume Changes of the Ipsilateral Lung

For the patients with the right lung as the ipsilateral lung, the initial mean volume of the ipsilateral lung was 1,296.71 cm^3^ (1,013.36–1,612.4 cm^3^), and the ipsilateral lung volume decreased during the first seven treatment fractions and then gradually increased from the 9th treatment fraction (see **Table 5**), with a maximum magnitude of no more than 10%. For the patients with the left lung as the ipsilateral lung, the initial mean volume of the ipsilateral lung was 593.4 cm^3^, ranging from 0 to 957.64 cm^3^. The mean lung volume of the ipsilateral lung tended to increase gradually during the course of treatment, and the magnitude of the increase was greater than that of the patients with the right lung as the ipsilateral lung, with a maximum of 25.83%.

**Table 5:** The volume changes of the ipsilateral lung.

	Right lung as ipsilateral lung		Left lung as ipsilateral lung	
	Volume (cm^3^)	Change (%)	Volume (cm^3^)	Change (%)
Plan	1,296.71 ± 1,76.21		593.40 ± 267.10	
f1	1,291.16 ± 177.30	−0.38 ± 3.76	621.19 ± 299.50	3.77 ± 9.05
f3	1,277.70 ± 170.39	−1.40 ± 2.73	620.78 ± 299.79	3.71 ± 8.87
f5	1,281.36 ± 140.8	−0.81 ± 4.27	619.35 ± 294.93	3.43 ± 8.67
f7	1,274.67 ± 162.77	−1.56 ± 3.64	644.26 ± 231.38	1.40 ± 11.25
f9	1,293.48 ± 141.85	0.25 ± 6.63	705.60 ± 192.77	4.64 ± 10.48
f11	1,316.72 ± 250.92	1.20 ± 9.96	866.61 ± 365.28	15.12 ± 24.47
f13	1,330.46 ± 245.82	2.31 ± 9.76	869.68 ± 360.62	16.70 ± 29.67
f15	1,352.42 ± 242.58	4.08 ± 9.75	905.03 ± 408.07	19.43 ± 29.74
f17	1,356.27 ± 243.68	4.35 ± 9.49	920.20 ± 371.24	26.09 ± 42.82
f19	1,344.87 ± 266.96	3.21 ± 9.39	916.90 ± 404.60	24.98 ± 48.35
f21	1,361.54 ± 282.73	4.37 ± 9.81	876.69 ± 403.22	15.41 ± 28.49
f23	1,395.06 ± 316.33	6.78 ± 12.29	895.77 ± 397.74	19.65 ± 34.03
f25	1,402.52 ± 312.84	7.31 ± 11.49	921.54 ± 397.78	25.08 ± 42.82
f27	1,425.83 ± 329.45	9.03 ± 12.85	930.12 ± 405.86	23.83 ± 29.19
f29	1,420.45 ± 351.65	8.35 ± 14.53	898.09 ± 429.95	16.52 ± 28.50

### The Dose Changes of PTV

All D_2_, D_98_, D_mean_, V_95_, and V_107_ of PTV decreased during the treatment course and decreased to the lowest value during the last two radiotherapy fractions (see **Table 6**). However, compared to the original plan, the magnitude of reduction of each indicator varied greatly. The reduction in D_2_ and D_mean_ was small during the course of radiotherapy, with the maximum reduction in D_2_ not exceeding 1% and the maximum reduction in D_mean_ reaching 3% by the 21st radiotherapy fraction and 3.61% by the end of radiotherapy. For V_95_, the reduction was greater than that of D_2_, reaching 4.01% by the 11th fraction and a maximum reduction of more than 8% at the end of radiotherapy. D_98_ and V_107_ showed the greatest reduction, both exceeding 3% at the 5th fraction, with maximum reductions of 25.33 and 16.91%, respectively. The TCP of the PTV reduced during the course of treatment and decreased to the lowest value at the last fraction.

**Table 6:** The dosimetry and biological changes of PTV.

	D_2_	Change (%)	D_98_	Change (%)	D_mean_	Change (%)	V_95_	Change (%)	V_107_	Change (%)	TCP	Change (%)
Plan	65.46 ± 0.78	−	58.66 ± 0.60	−	62.89 ± 0.46	−	99.25 ± 0.48	−	20.67 ± 13.32	−	0.6638 ± 0.0054	−
f1	65.45 ± 0.78	−0.02 ± 0.05	57.65 ± 1.93	−1.69 ± 2.94	62.75 ± 0.49	−0.22 ± 0.35	98.49 ± 1.45	−0.76 ± 1.30	20.08 ± 13.03	−2.54 ± 4.22	0.6621 ± 0.0058	−0.26 ± 0.41
f3	65.45 ± 0.79	−0.01 ± 0.03	57.29 ± 2.12	−2.09 ± 2.45	62.72 ± 0.46	−0.27 ± 0.30	98.31 ± 1.36	−0.95 ± 1.12	20.03 ± 12.90	−2.94 ± 3.40	0.6614 ± 0.0055	−0.35 ± 0.43
f5	65.45 ± 0.78	−0.02 ± 0.04	56.74 ± 2.83	−3.02 ± 4.03	62.61 ± 0.60	−0.45 ± 0.66	97.72 ± 2.46	−1.54 ± 2.30	19.84 ± 12.72	−3.89 ± 5.20	0.6602 ± 0.0075	−0.54 ± 0.85
f7	65.45 ± 0.78	−0.01 ± 0.04	56.52 ± 3.38	−3.35 ± 5.00	62.66 ± 0.50	−0.38 ± 0.49	97.90 ± 1.98	−1.36 ± 1.83	19.95 ± 12.63	−2.96 ± 4.94	0.6611 ± 0.0066	−0.40 ± 0.59
f9	65.45 ± 0.79	−0.02 ± 0.06	54.01 ± 10.62	−7.63 ± 18.03	62.45 ± 1.06	−0.71 ± 1.44	97.17 ± 3.39	−2.09 ± 3.34	19.79 ± 12.73	−4.50 ± 8.73	0.6572 ± 0.0167	−1.00 ± 2.27
f11	65.39 ± 0.80	−0.12 ± 0.30	50.58 ± 16.64	−13.77 ± 28.38	61.77 ± 2.61	−1.78 ± 3.99	95.27 ± 7.41	−4.01 ± 7.42	18.68 ± 13.35	−10.78 ± 21.33	0.6436 ± 0.0503	−3.04 ± 7.44
f13	65.39 ± 0.81	−0.12 ± 0.32	50.93 ± 16.78	−13.17 ± 28.61	61.77 ± 2.68	−1.80 ± 4.10	95.10 ± 8.09	−4.18 ± 8.13	18.79 ± 13.46	−10.27 ± 23.09	0.6430 ± 0.0511	−3.14 ± 7.55
f15	65.31 ± 0.91	−0.24 ± 0.60	50.97 ± 17.05	−13.16 ± 29.05	61.28 ± 4.85	−2.57 ± 7.57	93.94 ± 13.73	−5.35 ± 13.82	18.63 ± 13.93	−12.31 ± 31.39	0.6353 ± 0.0947	−4.31 ± 14.18
f17	65.32 ± 0.92	−0.22 ± 0.58	49.62 ± 17.75	−15.49 ± 30.20	61.35 ± 4.33	−2.46 ± 6.76	93.94 ± 12.32	−5.34 ± 12.39	18.86 ± 14.31	−11.40 ± 32.48	0.6343 ± 0.0874	−1.95 ± 5.69
f19	65.32 ± 0.92	−0.22 ± 0.60	48.56 ± 17.58	−17.27 ± 29.94	61.31 ± 4.24	−2.53 ± 6.61	93.49 ± 11.91	−5.8 ± 11.99	18.88 ± 14.43	−11.70 ± 32.68	0.6333 ± 0.0857	−4.60 ± 12.82
f21	65.34 ± 0.93	−0.19 ± 0.57	46.07 ± 20.67	−21.49 ± 35.21	60.96 ± 4.42	−3.08 ± 6.92	92.55 ± 12.61	−6.74 ± 12.72	19.14 ± 14.32	−10.70 ± 31.00	0.6259 ± 0.0893	−5.72 ± 13.38
f23	65.32 ± 0.93	−0.22 ± 0.59	45.85 ± 20.59	−21.66 ± 35.17	60.98 ± 4.31	−3.05 ± 6.75	92.79 ± 12.18	−6.50 ± 12.30	18.79 ± 14.47	−11.98 ± 33.04	0.6278 ± 0.0857	−5.43 ± 12.83
f25	65.32 ± 0.93	−0.23 ± 0.59	45.52 ± 20.46	−22.42 ± 34.84	60.99 ± 4.20	−3.04 ± 6.57	92.56 ± 11.92	−6.73 ± 12.03	18.81 ± 14.58	−12.03 ± 32.90	0.6267 ± 0.0846	−5.60 ± 12.68
f27	65.27 ± 0.96	−0.30 ± 0.65	43.81 ± 21.36	−25.33 ± 36.41	60.63 ± 4.35	−3.61 ± 6.79	91.01 ± 13.17	−8.29 ± 13.31	18.15 ± 14.97	−16.91 ± 35.98	0.6237 ± 0.0845	−6.06 ± 12.63
f29	65.29 ± 0.97	−0.27 ± 0.64	44.25 ± 20.80	−24.56 ± 35.43	60.71 ± 4.33	−3.48 ± 6.74	91.17 ± 1 3.06	−8.12 ± 13.2	18.42 ± 14.86	−15.75 ± 35.09	0.6237 ± 0.0852	−6.05 ± 12.72

### Change in Total Lung Dose With Treatment Fractions

The V_5_, V_10_, V_20_, V_30_, and V_40_ of the total lung increased gradually during the treatment course and increased to the highest value at the end of radiotherapy. As shown in **Table 7**, for the same fraction, the increase in each parameter increased sequentially, from V_5_, V_10_, V_20_, V_30_ to V_40_, and compared to the original plan, the magnitude of the increase in each parameter was large. All of the parameters increased by more than 4% by the 1st fraction, and essentially the maximum magnitude exceeded 20% by the last fraction. Similarly, the NTCP of total lung increased also gradually during the treatment course and increased from 0.0941 to the highest value 0.1302 at the end of radiotherapy.

**Table 7:** The dosimetry and biological changes of total lung.

	V_5_	Change (%)	V_10_	Change (%)	V_20_	Change (%)	V_30_	Change (%)	V_40_	Change (%)	NTCP	Change (%)
Plan	32.79 ± 6.74	–	24.85 ± 5.95	–	18.19 ± 4.51	–	14.32 ± 4.27	–	10.92 ± 4.11	–	0.0941 ± 0.1427	–
f1	34.01 ± 7.77	4.37 ± 17.99	25.86 ± 6.57	5.38 ± 19.96	18.98 ± 5.02	5.93 ± 21.82	14.93 ± 4.47	6.64 ± 25.42	11.40 ± 4.13	10.92 ± 4.11	0.0987 ± 0.1416	12.17 ± 43.91
f3	33.87 ± 7.75	3.94 ± 17.74	25.75 ± 6.48	4.99 ± 19.66	18.89 ± 4.79	5.67 ± 21.47	14.82 ± 4.19	6.43 ± 24.90	11.29 ± 3.80	11.40 ± 4.13	0.0975 ± 0.1415	10.90 ± 42.47
f5	33.59 ± 7.99	2.99 ± 18.38	25.51 ± 6.68	3.94 ± 20.34	18.63 ± 4.84	4.37 ± 22.31	14.58 ± 4.15	5.18 ± 25.30	11.07 ± 3.69	11.29 ± 3.80	0.0947 ± 0.1417	6.82 ± 39.371
f7	33.89 ± 7.56	4.20 ± 17.78	25.76 ± 6.34	5.21 ± 19.52	18.91 ± 4.55	6.33 ± 21.43	14.84 ± 3.94	7.70 ± 25.27	11.30 ± 3.63	11.07 ± 3.69	0.0979 ± 0.1411	12.25 ± 40.97
f9	34.30 ± 7.43	5.82 ± 19.16	26.24 ± 6.20	7.78 ± 21.59	19.47 ± 4.31	11.12 ± 27.8	15.49 ± 3.65	16.21 ± 40.78	11.96 ± 3.43	11.30 ± 3.63	0.0990 ± 0.1404	16.65 ± 48.04
f11	35.21 ± 6.70	9.36 ± 20.96	27.17 ± 5.64	12.59 ± 24.99	20.51 ± 4.04	19.45 ± 41.45	16.56 ± 3.66	29.03 ± 70.37	13.01 ± 3.71	11.96 ± 3.43	0.1030 ± 0.1344	29.98 ± 67.41
f13	35.56 ± 6.64	10.51 ± 21.09	27.52 ± 5.67	14.08 ± 25.18	20.89 ± 4.19	21.51 ± 41.37	17.03 ± 3.91	32.24 ± 70.36	13.42 ± 3.92	13.01 ± 3.71	0.1040 ± 0.1301	32.68 ± 66.96
f15	35.93 ± 6.87	11.62 ± 21.56	27.97 ± 5.88	15.86 ± 25.80	21.39 ± 4.40	24.25 ± 41.91	17.46 ± 4.02	35.08 ± 69.97	13.84 ± 4.02	13.42 ± 3.92	0.1078 ± 0.1322	36.78 ± 63.77
f17	36.18 ± 7.26	12.34 ± 22.53	28.20 ± 6.12	16.76 ± 26.30	21.69 ± 4.66	25.80 ± 41.71	17.77 ± 4.34	37.20 ± 70.05	14.19 ± 4.32	13.84 ± 4.02	0.1048 ± 0.1329	32.77 ± 75.83
f19	36.50 ± 7.55	13.38 ± 23.97	28.57 ± 6.83	18.02 ± 28.50	22.15 ± 5.83	27.45 ± 42.55	18.29 ± 5.58	39.39 ± 69.23	14.74 ± 5.39	14.19 ± 4.32	0.1225 ± 0.1342	37.23 ± 65.68
f21	36.67 ± 7.78	13.92 ± 24.92	28.76 ± 7.03	18.83 ± 29.73	22.27 ± 6.05	28.18 ± 43.9	18.36 ± 5.87	39.73 ± 70.74	14.73 ± 5.73	14.74 ± 5.39	0.1093 ± 0.1290	39.82 ± 71.42
f23	36.73 ± 7.96	14.19 ± 25.92	28.89 ± 7.28	19.44 ± 31.07	22.48 ± 6.30	29.35 ± 44.95	18.63 ± 6.03	41.70 ± 71.36	15.08 ± 5.78	14.73 ± 5.73	0.1014 ± 0.1326	28.79 ± 85.16
f25	36.83 ± 7.90	14.40 ± 25.25	29.01 ± 7.18	19.82 ± 30.24	22.63 ± 6.14	30.10 ± 44.02	18.79 ± 5.84	42.92 ± 70.51	15.23 ± 5.59	15.08 ± 5.78	0.1119 ± 0.1291	44.17 ± 75.47
f27	36.86 ± 7.87	14.53 ± 25.30	29.08 ± 7.14	20.23 ± 30.32	22.72 ± 6.07	30.79 ± 43.96	18.91 ± 5.71	44.14 ± 70.31	15.39 ± 5.42	15.23 ± 5.59	0.1302 ± 0.1349	51.15 ± 89.59
f29	36.75 ± 7.89	14.23 ± 25.45	28.96 ± 7.24	19.78 ± 30.69	22.54 ± 6.31	29.79 ± 44.76	18.75 ± 5.96	42.82 ± 71.10	15.26 ± 5.63	15.39 ± 5.42	0.1135 ± 0.1291	46.95 ± 76.09

### Summary of Changes in Dosimetry and Geometry

As shown in **Tables 2**, **3**, the GTV tended to gradually decrease, and the COM of the GTV shifted mediastinally during the course of radiotherapy. Therefore, D_2_, D_98_, and D_mean_ of the PTV decreased gradually (**Table 6**). **Figure 1** shows that four patients had complete regression, six patients had partial regression, and six patients had almost no change during radiotherapy, which meant that the atelectasis regressed in half of the patients, which increased the volume of the ipsilateral lungs. Since most of the lung cancer patients in this study had central lung cancer, the atelectasis was located next to the tumor, so the increased lung volume was also located adjacent to the tumors, which increased the dose to lung tissue.

## Discussion

In this paper, we retrospectively studied lung cancer patients with atelectasis during radiotherapy to explore geometric and dosimetric changes in the target and lung tissues. To our knowledge, this is the first comprehensive study of targets and lung tissue in patients with atelectasis. Currently, studies on atelectasis radiotherapy are few and are generally retrospective (5, 11, 12), mainly due to the limited number of patients with atelectasis and the large number of images to be acquired.

Usually, central lung cancer is accompanied by atelectasis and obstructive inflammation. Due to tracheal obstruction, ventilation and drainage disorders, reduced effective lung volume and local inflammation, patients often experience severe asthma, chest tightness, dyspnea, fever, and other symptoms. Therefore, the risk of radiotherapy for these patients with atelectasis is higher than that for patients with non-atelectasis lung cancer, and the complications were also more. Atelectasis may undergo regression or expansion during radiotherapy, which may cause major anatomical changes in the tumor. Therefore, image guidance radiotherapy technology and adaptive radiotherapy are necessary. At present, there is currently a lack of guidelines for adaptive radiotherapy for atelectasis. From some of the few studies related to atelectasis, it is known that the density and quality of atelectasis will change, and this change will generate a dosimetry effect on the surrounding normal tissues (5, 11, 12). However, it is not clear how much the regression or expansion of atelectasis affected the geometry and dosimetry of the tumor and lung. Also, the time of the regression or expansion of atelectasis is still unknown. These are the main reasons for this study. To our knowledge, this is the first comprehensive study of targets and lung tissues in patients with atelectasis.

Most of the patients studied in this paper had central lung cancer, and the primary tumor was located near the hilar, so this may be the main reason why the COM and the boundary of the GTV moved to the center of the mediastinum in this study. Other reasons may be related to the location of the atelectasis. The location of atelectasis in this study was mainly in the upper and lower lung lobes (17/18 patients). For the three patients with peripheral lung cancer, the location of the tumor was very different, but the degree of atelectasis regression did not vary and had little influence on the statistical results of this paper. Therefore, we did not explore the differences between the central and peripheral lung cancer groups in this study, but further study of this issue will be conducted.

**Table 2** shows that the volume of GTV during radiotherapy gradually decreased, and the larger the volume was, the greater the reduction. However, there were eight patients whose volume increased slightly during the first fractions. Through the observation of CBCT/CT images, it was hypothesized that the increase in GTV volume might be related to tumor inflammation and edema.

Previous results showed that, compared with the shifts of GTV in patients without atelectasis, the shifts of GTV in patients with atelectasis were larger, and for 58% of patients they were more than 1 cm (12). Similar results were observed in this study; 50% of patients (8/18) with a shift of more than 1 cm were also found.

The regression or expansion of the atelectasis reduced the dose coverage rate of the target, but the change in the dose index of the target was very different. D_2_ and D_mean_ decreased slightly (0.3 and 3.48%), and D_98_ (25.33%) and V_107_ (16.91%) decreased significantly (25.33 and 16.91%, respectively). This meant that due to the shift of the GTV, 25.33% of the target dose would be seriously insufficient when using the original plan, and 16.91% of the high doses were delivered outside of the tumors; as a result, the TCP also reduced. For patient 4, patient 10 and patient 22, the degree of atelectasis regression was larger, resulting in a larger GTV shift in the three-dimensional direction, up to 4.73 cm. As a result, D_98_ decreased by more than 50%, and V_107_ decreased by more than 25%. According to Christopher et al. (11), midtreatment alignment based on the carina, rather than on the bone, led to a smaller dose difference during the follow-up than at baseline. In this study, the subject was aligned *via* bone; if registration based on carina or soft tissue is adopted, the COM, boundary offset, and dosimetric changes for the target may be smaller.

Generally, the regression or expansion of atelectasis may cause an increase or decrease in lung volume. The larger the amount of regression or expansion, the larger the increase or decrease in lung volume. As shown in **Table 5**, the volume of the ipsilateral lung increased gradually throughout the radiotherapy course. One of the reasons may be tumor regression, but the main reason may be that most patients with atelectasis had regression (12 cases). For one patient in this study with left whole atelectasis, the atelectasis gradually regressed, the dose to the total lung increased, and the NTCP of total lung decreased gradually throughout the whole course of radiotherapy.

Central lung cancer is close to the spinal cord, large blood vessels, and bronchi, and other important organs are at risk. If high doses are delivered to these organs at risk, the risk of complications may be increased. Adaptive planning may benefit these patients (5, 22–24). In our center, each CBCT image collected by every patient with atelectasis was evaluated. At present, there are no corresponding adaptive planning guidelines, and we are conducting relevant adaptive research for atelectasis radiotherapy. The results showed that the resolution or expansion of atelectasis may occur during any fraction of radiotherapy. Therefore, we suggest that CBCT should be collected at least every other day for evaluation. This paper focused on the geometric and dosimetric changes in the target and lung tissue in patients with atelectasis during radiotherapy. Hierarchical research is needed to determine the relevant changes in different types of atelectasis, which could provide a more accurate reference for future adaptive treatment clinical applications.

Although atelectasis often occurs in central lung cancer (8), few atelectasis cases occur in peripheral lung cancer (25). As seen from the results of this paper, we found that there are significant differences between the two types of lung cancer and their effects on the geometry and dose to the tumors and the OARs. Even for central lung cancer, the location of atelectasis varies. The COM and boundary shift of the tumor are also different when regression or expansion occurs. Thus, stratifying patients with atelectasis warrants further investigation.

Considering that atelectasis is usually located near the GTV, CBCT images cannot completely distinguish atelectasis and GTV, and other clearer imaging methods (such as CT or MRI) are needed to distinguish atelectasis from GTV (26). Therefore, the volume or boundary changes of atelectasis were not included in this paper.

This study also had some limitations. One limitation of this study was the low number of patients. The main reason is that fewer than 35% of atelectasis cases present at the start of lung cancer radiotherapy treatment (3–7), and some patients terminate radiotherapy because of radiation reactions. Our center will continue to enroll patients with atelectasis for further study. Second, the contrast resolution of the CBCT images was poor, and the electron density was inaccurate (5, 7, 13), which made it challenging to clearly identify tumors from the atelectatic regions. Frequent CBCT scans may also increase the risk of secondary cancer, and magnetic resonance imaging may be a better choice.

## Conclusions

In conclusion, for most patients with atelectasis, atelectasis gradually regressed, and the GTV gradually shifted to the center of the mediastinum, resulting in a lower dose in the target volume. The volume of the ipsilateral lung increased, and the dose to the lung tissue increased. Since resolution or expansion of atelectasis may occur during any fraction during radiotherapy, we propose an evaluation with CBCT at least every two fractions.

## Data Availability Statement

The raw data supporting the conclusions of this article will be made available by the authors, without undue reservation.

## Ethics Statement

The studies involving human participants were reviewed and approved by the ethics committee in the Shanghai chest hospital. The patients provided written informed consent to participate in this study.

## Author Contributions

HC analyzed the data and wrote the manuscript. All authors participated in the design of the presented study, reviewed the manuscript prior to its publication. All authors contributed to the article and approved the submitted version.

## Funding

This work was sponsored by the Interdisciplinary Program of Shanghai Jiao Tong University (Grant No. YG2019ZDB07) and Nurture projects for basic research of Shanghai Chest Hospital (Grant No. 2019YNJCM05).

## Conflict of Interest

The authors declare that the research was conducted in the absence of any commercial or financial relationships that could be construed as a potential conflict of interest.

## Abbreviations

CBCT, cone beam computed tomography; COM, center of mass; TCP, tumor control probability; NTCP, normal tissue complication probability; GTV, gross tumor volume; OAR, organ at risk; PET, positron emission tomography; PTV, planning target volume; LR, left-right; AP, anterior–posterior; SI, superior–inferior; HU, Hounsfield unit.
